# Successful twin pregnancy in a patient with lupus erythematosus on hemodialysis: a case report and literature review

**DOI:** 10.1186/s12882-025-04491-8

**Published:** 2025-10-13

**Authors:** Sebastian Heibel, Helene Rohde, Susanne Marek, Kirsten de Groot

**Affiliations:** 1 Department of Internal Medicine III (Nephrology and Dialysis, Hypertensiology, Rheumatology), Sana Klinikum Offenbach GmbH, Starkenburgring 66, Offenbach am Main, 63069 Germany; 2 KfH Kidney Center Offenbach, KfH Kuratorium für Dialyse und Nierentransplantation e.V., Starkenburgring 70, Offenbach am Main, 63069 Germany; 3 Department of Gynecology and Obstetrics, Sana Klinikum Offenbach GmbH, Starkenburgring 66, Offenbach am Main, 63069 Germany

**Keywords:** Twin pregnancy, Hemodialysis, End-stage renal disease, Lupus erythematosus, Case report

## Abstract

**Background:**

Pregnancy rates in women on hemodialysis are low but increasing due to improvements in nephrological and obstetrical care. All pregnancies in dialysis patients qualify as high-risk pregnancies, but courses and outcomes of pregnancy vary depending on the underlying cause of renal failure. While more information about best practice in treatment of pregnant dialysis patients has accumulated recently, there are few cases described with systemic lupus erythematosus (SLE) as underlying disease and there is almost no information about twin pregnancies in this cohort.

**Case presentation:**

We report the case of a successful twin pregnancy in a 30-year old hemodialysis patient with SLE who had repeatedly miscarried before. Thanks to her remaining own diuresis the required augmentation of dialysis dose was moderate with 25–27.5 h/week, leading to largely physiological blood urea levels. In the course of pregnancy, the patient experienced severe preeclampsia, so anticipated C-section delivered both twins at 34 + 1 weeks of gestation. Both newborns were appropriate for gestational age. The boy was clinically stable but the girl initially needed intensive care. In the postpartum period the mother developed hypertensive encephalopathy and status epilepticus treated successfully. Both mother and children are well and do not suffer from any lasting damage acquired during pregnancy.

**Conclusions:**

Pregnancy in dialysis patients is feasible even in challenging conditions as the combination of end-stage renal disease, SLE and twin pregnancy. Our case demonstrates a possible treatment regimen in a special clinical situation. By changes in medication and augmented dialysis dose appropriate to the specific individual extent of uremia, we achieved a near normal duration of pregnancy. More data on rare conditions such as twin pregnancies on dialysis with underlying autoimmune disorders are necessary to give firm advice.

## Introduction

In women on maintenance dialysis, spontaneous conception is a rare event. The main reasons for limited fertility in end-stage renal disease (ESRD) are uremia-induced sexual dysfunction and endocrine alterations causing anovulatory cycles, but psychological factors also play a role [1, 2]. However, pregnancy rates in dialyzed women have risen in the last decades, reflecting advancements in dialysis, nephrological and obstetric care, but also in counseling practices [3]. Still, the risks for mother and fetus are elevated and the live birth rate is considerably reduced compared to pregnant women without chronic renal disease [4, 5]. Data regarding the best dialysis regimen and scheme of clinical follow-ups during dialysis patients’ pregnancies are limited. Besides, outcome seems to differ depending on the different underlying causes of renal failure, ranking lupus-associated glomerulonephritis (LN) as a particularly disadvantageous underlying disease [6, 7].

Unfortunately, systemic lupus erythematosus (SLE) mostly affects young women in childbearing age. LN affects up to 50–75% of patients, of which 10–25% finally need renal replacement therapy [8]. Pregnancies in SLE itself already qualify as risk pregnancies and often accompany both maternal and neonatal complications as flares of disease activity, preeclampsia, fetal loss and preterm birth, fetal congenital heart block and neonatal lupus. However, the overall prognosis of SLE pregnancies has also improved over time [9, 10].

The combination of ESRD with dependence on maintenance dialysis, underlying SLE and twin pregnancy is very rare and severely increases the risk for an unfavorable pregnancy outcome.

We report on a patient on maintenance hemodialysis (HD) due to LN who fell spontaneously pregnant with twins that could be delivered safely by C-section at 34 + 1 weeks of gestation (WOG).

## Case presentation

A 30-year-old woman with SLE undergoing maintenance HD for almost 2 years was referred to our hospital semi-inpatient dialysis unit, being 16 weeks pregnant, for intensification of HD and monitoring.

Her SLE initially manifested 5 years earlier and was confirmed histologically 2 years after manifestation during an episode of acute renal failure due to LN class III as per the ISN/RPS classification. Serology showed antinuclear antibodies (ANA) and anti-dsDNA antibodies, antiphospholipid syndrome was ruled out. Having stopped taking the prescribed mycophenolate (MMF) and prednisone, in the following year, she had developed full-blown nephrotic syndrome with proteinuria of 20.1 g/g creatinine and acute on chronic renal failure (serum creatinine 6.3 mg/dl). Kidney re-biopsy revealed a partially diffuse and partially membranous lupus glomerulonephritis with marked tubulointerstitial damage involving approximately 35–40% of the cortex. As the sample was considered non-representative (only six glomeruli), a reliable distinction between ISN/RPS class IV and class V could not be made. Despite treatment with methylprednisolone, MMF, rituximab and therapeutic plasma exchange, she finally needed renal replacement therapy and started HD 3 times/week via an arteriovenous fistula.

Being on maintenance HD for 7 months, she found herself 5 weeks pregnant while on MMF and methylprednisolone. Termination was recommended due to the teratogenicity of MMF and a high risk of miscarriage due to the drug and uremia. The patient insisted on continuation of the pregnancy, but, despite stopping MMF immediately, had an early spontaneous abortion. With an ongoing desire to conceive she did not resume MMF, but had another spontaneous pregnancy and early abortion several months later. Following this, the patient continued on methylprednisolone monotherapy, during periods without rheumatologic follow-up.

At the time of conception of a new spontaneous pregnancy, the patient showed no signs of disease activity. Anti-dsDNA antibody levels measured externally were mildly elevated at 19 IU/mL (normal < 13 IU/mL), and ANA titers were positive at 1:640 with a nucleolar-homogeneous fluorescence pattern, complement levels (C3/C4) were within the normal range. In the first trimester the patient remained clinically almost asymptomatic and only reported mild Raynaud’s symptoms at a new rheumatologic follow-up-visit at 12 WOG. At this point, laboratory evaluation showed no hematologic abnormalities, no complement consumption, and no systemic inflammation. The ANA titer was 1:320, anti-dsDNA antibodies were 27 IU/mL (normal < 20 IU/mL). Antiphospholipid antibodies were negative, while anti-SSA antibodies were detected. Hydroxychloroquine was recommended as maintenance therapy, but was finally started only at 16 WOG (see below). Given the high-risk pregnancy and the presence of anti-SSA antibodies, referral to a center capable of providing fetal and neonatal intensive monitoring was recommended due to the risk of congenital heart block.

At her referral to our dialysis unit in her 16th WOG of the current pregnancy - this time with twins - she had already gained approximately 5 kg of weight, having been of normal weight at the beginning of pregnancy (body mass index 21.1). Her medication consisted of methylprednisolone 6 mg, ASA 100 mg, Magnesium 40 mg daily, cholecalciferol 20,000 IU every 3 weeks plus calcitriol 0,5 µg 3times/week orally. Erythropoietin alpha 5,000 IU IV as erythrocyte stimulating agent (ESA) and water-soluble vitamins (Vitarenal^®^) 2 capsules were delivered after every HD, IV iron sucrose 100 mg twice a week. Folic acid 5 mg/day was paused at the time.

We intensified HD, which had been augmented in the outpatient dialysis from 3/week 4.5 h to 3–4/week 6 h to a treatment time of 5/week 5 h and extended duration to 5.5 h per HD from 19 WOG, thus achieving a total dialysis time of 25–27.5 h weekly. As the patient refused a further increase of HD time and had a good residual renal function serum urea levels could be reduced to normal values by this regime (mean ± standard deviation: pre-dialysis 39,88 ± 15,91 mg/dL, post-dialysis 7,50 ± 1,06 mg/dL). To maintain this extent of uremia reduction, dialysate flow was increased from 500 mL/min at the time of referral to 600 mL/min from 22 WOG. We used a 1.7 m²-Polyamix^®^-High-Flux Dialyzer (Polyflux 170 H^®^, Gambro, Hechingen, Germany) with average blood flow 260–300 mL/min, unfractionated heparin for anticoagulation and chose a dialysate composition with potassium 4 mmol/L, calcium 1,5 mmol/L, sodium 136–140 mmol/L, bicarbonate 32 mmol/L and glucose 1 g/L. During the course of the intensified dialysis regimen, the bicarbonate concentration in the dialysate could be reduced to 30 mmol/L, while maintaining serum bicarbonate levels within the target range of 20 to 26 mmol/L. Thanks to good own urine production, no correction of volume status via ultrafiltration was needed and blood pressure could be held within the normal range until the last week of gestation without antihypertensives. Estimated dry weight was increased about 12 kg from 16 WOG to the date of delivery.

**Fig. 1:**
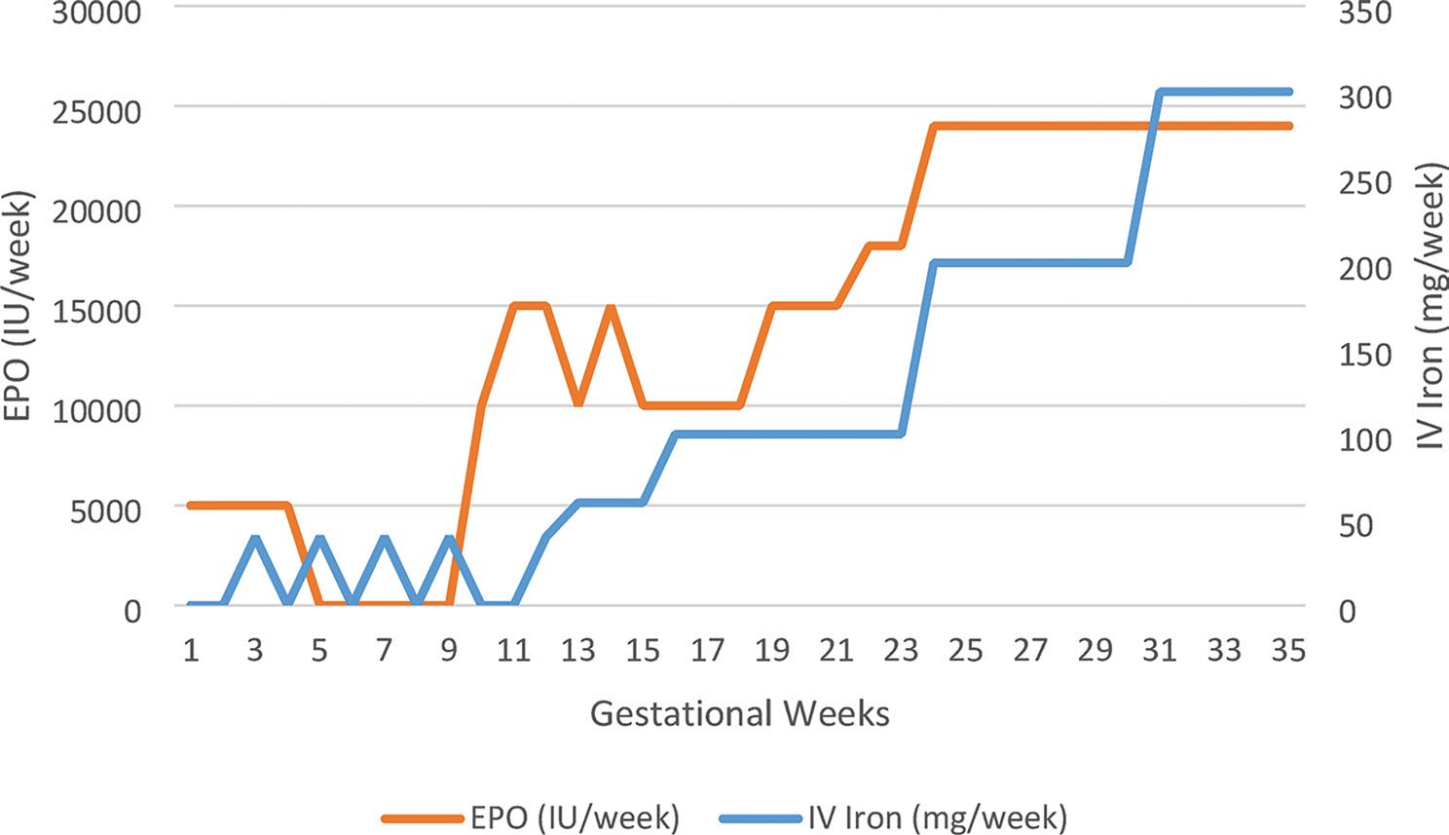
Erythropoietin (EPO) and IV Iron requirements during pregnancy. Weekly iron dose (mg) and EPO dose (IU) are shown in relation to gestational age. Note: Data from gestational weeks 1 to 16 were obtained from the external dialysis center where the patient was treated before referral to our center at 16 WOG

The initial medication was continued, administration of folic acid (5 mg/d) resumed. We added Hydroxychloroquine 200 mg/d for immunomodulation, prevention of maternal SLE flares and neonatal lupus of the unborn twins, as the patient had SSA antibodies. Pantoprazole was given for heartburn. During the following weeks, worsening anemia made us raise the ESA dose 1.5-1.6-fold (to erythropoietin alpha 3 × 8000 IU/week) compared to the dose administered at 16 WOG, also the need for IV iron rose from 100 mg/week to 300 mg/week to achieve hemoglobin levels between 10 and 11 g/L (Fig. 1). Notably, significantly lower doses of both ESA and IV iron had been sufficient prior to 16 weeks of gestation at the referring outpatient dialysis center, underscoring the marked increase in requirements.

We recommended our patient a balanced diet rich in high-quality proteins with normal intake of potassium, phosphate and other electrolytes. Phosphate binders were not needed as serum phosphate level was normal (0.9–1.22 mmol/L) under the intensified dialysis regimen during the entire pregnancy. Other electrolytes could be held in normal range throughout the whole pregnancy as well (serum potassium 4.1–5.0 mmol/L, serum calcium 2.13–2.36 mmol/L). The vitamin D supplementation dose did not require adjustment during pregnancy, and parathyroid hormone (PTH) levels remained within the recommended target range of two- to sixfold elevation, with values around 20 pmol/L.

Obstetric ultrasound showed a dichorionic, diamniotic twin pregnancy. Fetal growth was monitored by serial ultrasound scans every 2–3 weeks. Both fetuses showed biometric values normal for gestational age and no congenital malformations. Amounts of amniotic fluid were unremarkable at any time, polyhydramnios did not occur. The placentas did not show any abnormalities. Fetomaternal Doppler results including the umbilical artery and the middle cerebral artery as well as the maternal uterine artery were within normal range.

Proteinuria was stable from 2.08 g/24 h (17 WOG) until at least 28 WOG (2.16 g/24 h). At 32 WOG, we noticed an increase to 3.92 g/24 h whereas blood count showed no thrombocytopenia and blood pressure was < 140/90 mmHg.

From 34 WOG blood pressure peaked at 180/100 mmHg, thus the patient was hospitalized at 33 + 4 WOG with suspected severe preeclampsia. At this point, the estimated weight of the fetuses was 1872 g and 2037 g, both in cephalic presentation and without pathological findings concerning amniotic fluid amounts, placentas and Doppler values. Maternal uterine artery Doppler examination did not show any notching or high resistance typical for preeclampsia. The sFlt-1/PlGF ratio, a biomarker predictive of preeclampsia [11], was normal the day of admission. Alternative causes of hypertension, such as increased lupus activity or thrombotic microangiopathy (TMA), were ruled out: There were no fever, arthralgia, serositis or cytopenia, complement levels were normal. Likewise, there was absence of thrombocytopenia and hemolytic anemia, elevated (rather than decreased) haptoglobin levels, and no schistocytes could be detected.

We administered methyldopa 250 mg 3/day as antihypertensive medication, stopped the intake of ASA at 33 + 4 WOG and gave a single course of antenatal corticosteroids to accelerate fetal lung maturation.

Because of the heavily increased risk of developing preeclampsia, a planned C-section was performed at 34 + 1 WOG. Both newborns’ weight and size were just appropriate for gestational age (girl: 1830 g, 43 cm; boy: 1960 g, 42 cm). The second-born child (boy) was stable with normal APGAR scores 9-9-8; the first-born child (girl) was transferred to the NICU due to respiratory insufficiency and APGAR score 6-7-7. After support with a cPAP device for some hours and a single phototherapy for neonatal hyperbilirubinemia her medical condition improved and she could be moved to the general neonatal ward the following day.

Six days postpartum, despite intensified antihypertensive therapy with methyldopa, nifedipine, and -following cessation of breastfeeding - the addition of ramipril, the patient developed a hypertensive crisis during hemodialysis, with blood pressure rising to 230/130 mmHg in the absence of volume overload. She subsequently experienced a severe headache and visual disturbances, progressing to status epilepticus, which required intensive care management and termination with 2 mg of lorazepam. A cranial CT scan ruled out intracerebral hemorrhage as well as ischemic stroke. However, bilateral occipital and right parietal hypodensities were observed, consistent with posterior reversible encephalopathy syndrome (PRES) as the most likely diagnosis. Anticonvulsant therapy with levetiracetam was initiated. After three days of further antihypertensive escalation—including dihydralazine, amlodipine, intravenous urapidil, and nitroglycerin—the patient was transferred to the general ward for transition to oral antihypertensives and final blood pressure adjustment. At her discharge 18 days after delivery, the antihypertensive medication consisted of ramipril 5 mg 2/day, bisoprolol 5 mg 2/day, dihydralazine 50 mg 2/day and methyldopa 500 mg 4/day. Anticonvulsant therapy was continued.

The patient switched to peritoneal dialysis (PD) five months after childbirth for better compatibility with childcare and now awaits kidney transplantation. Her good residual renal function was preserved with urine output completely equaling fluid intake (1.1–2.0 L/24 h) and no need for ultrafiltration via dialysis. Creatinine levels under maintenance HD 3 times/week remained comparable before and after pregnancy. Both twins are in good condition and show adequate development.

### Discussion and conclusions

Pregnancies in dialysis patients remain uncommon and entail a high risk of fetal and an elevated risk of maternal morbidity and mortality [12]. However, pregnancy rates have risen from 0.54 to 3.3 per 1000 patients-years until the end of the first decade of the 21. century [13] and seem to further increase [3, 12]. Unfortunately, the low pregnancy rates reported are accompanied by an even lower live birth rate. While in the 1990s, infant survival rate reported was only 42% [14], recent studies observed rising live birth rates up to 85%, in particular in HD patients [15, 16].

A better residual renal function with preserved urine production is associated with a better outcome of pregnancy [17] and, as in our case, allows liberalized volume management via dialysis. PD patients often preserve better residual renal function [15], but conception rate is lower in this population as compared to HD patients [18]. Besides, possible complications after conception and a higher rate of babies small for gestational age in PD patients led to the recommendation to switch from PD to HD at the time of conception [19] or even when trying to become pregnant [4].

Due to irregular menstruation and hormonal state in ESRD, detection of pregnancy is often delayed, in the mean to 16.5 WOG. This can bear deleterious effects on fetal development, e.g. due to continuation of teratogenic medication – as happened to our patient previously – or delay of a beneficial intensification of dialysis [20].

Augmentation of HD time from < 18 h/week to 37–56 h/week during gestation was related to a significant increase in live birth rate (85% vs. 48%), gestational age at delivery (38 vs. 28 weeks) and median birth weight (2.6 kg vs. 1.8 kg) [21], but means a high burden for the patient. Some authors consider it sufficient raising the dialysis dose to at least 20 h/week to achieve a serum BUN level below 50 mg/dL, but state a further augmentation to 24 h/week as beneficial [22, 23]. We chose a HD dose (25 h/week to 27.5 h/week) which led to a good outcome of 100% live birth rate in another setting [24]. Evidently, the extent of maternal uremia rather than the duration of dialysis itself largely determines the pregnancy outcome in dialysis patients. Serum BUN levels and both birth weight and gestational age were negatively correlated: BUN levels < 48–49 mg/dL (**≈** serum urea 102 mg/dL) corresponded to a birth weight >1500 g and a gestational age >32 WOG [25]. Maternal uremia is thought to cause a fetal solute diuresis resulting in polyhydramnios, which itself can lead to premature birth [26], so there is a recent trend to target even lower BUN levels < 35 mg/dL [6]. In our case, most blood tests showed completely normal pre-dialysis serum urea values of 20–48 mg/dL and polyhydramnios did not occur. A similar reduction of uremia by nocturnal dialysis of 48 ± 5 h/week could achieve almost full term pregnancies [27]. We see both birth weight and gestational age in our case acceptable compared to the data reported in *non*-twin dialysis pregnancies.

Maternal risks of pregnancy in dialysis patients include uncontrollable hypertension up to (pre)eclampsia, loss of residual urine production and, in autoimmune disorders, flares of the underlying disease.

As frequently observed, methyldopa, the drug of choice for hypertension treatment in pregnancy [28], was not sufficient in our case. The medication can be expanded, e.g. by beta blockers as metoprolol or calcium channel blockade such as nifedipine, but teratogenic medication must be avoided.

Adjustment of dry weight during pregnancy is essential to minimize hypertension to post dialysis blood pressure readings < 140/90 mmHg and avoid hypertensive pregnancy disorders, but it can be challenging, because placental hypoperfusion due to intradialytic hypotension also must be prevented. Worth noting, the estimated weight gain (with single fetus) is 1–1.5 kg in the first trimester, but 0.45 to 1 kg *per week* in the second and third trimester [29].

In our case, we considered (pre-)eclampsia as differential diagnosis and reason for the postpartum status epilepticus. Preeclampsia as gestational hypertension accompanied by proteinuria [30] occurs in up to 40% of CKD patients’ pregnancies [20]. It can turn into eclampsia, a severe complication defined as a seizure [31] that can also occur in the postpartum period [32]. Numerous risk factors for preeclampsia include primiparity, CKD, autoimmune diseases and multifetal gestation [30].

Indeed, variations in blood pressure, preeclampsia and autoimmune disorders including SLE are known risk factors also associated with PRES, a neurological syndrome including various clinical presentations such as headache, focal neurological deficits, encephalopathy, visual disturbances and seizures [33]. Corresponding to our case, radiological imaging of PRES shows vasogenic edema especially in the parietal and occipital cerebral regions [34].

Our patient had a high risk for both preeclampsia and PRES and both entities share similar pathophysiology and symptoms [35]. When our patient presented with hypertension and rising proteinuria, the sFlt-1/PlGF ratio was not elevated, as would be expected in preeclampsia, and the maternal uterine artery Doppler was normal. Therefore, we interpreted the rise in blood pressure rather as pregnancy-induced hypertension than as preeclampsia. Considering the postpartum neuroimaging, PRES seems the more probable cause for the status epilepticus, but a firm conclusion remains difficult. A crucial part of the diagnostic workup in this context was to rule out a SLE flare or thrombotic microangiopathy; however, in our case, there was no clinical or serological evidence supporting either condition.

As anemia of pregnancy increases risk for low birthweight and preterm delivery [36], we aimed the target hemoglobin level in pregnancy 10,0–11 g/L for women with normal renal function and on dialysis [4, 19]. The administration of ESA (typically at 1.5- to 2-fold the pre-pregnancy dose, as in our case) and intravenous iron is considered safe for both mother and fetus [37].

The intensified HD schedule allows liberalization of diet. The recommended protein intake for patients on maintenance HD of 1,2 g/kg of ideal body weight per day is the minimum recommendation for pregnant dialysis patients [20], some authors also suggest unrestricted or even high protein diets with protein intake up to 1.5–1.8 mg/kg/day [22, 35]. Dialysate potassium was typically raised to 4 mmol/L, bicarbonate substitution was reduced, phosphate binders were not needed. Calcium-phosphate-metabolism was monitored carefully to ensure adequate development of the fetal skeleton and avoid possible negative effects [38] on maternal bone health. Doubling the supplementation of water soluble vitamins including folic acid in pregnancy is common practice as elimination via frequent HD is augmented, yet there are no precise dosing recommendations [4, 39].

Only few reports exist dealing with twin pregnancy in dialysis patients [40–42]. Even fewer focused the impact of HD-dependent ESRD due to SLE on pregnancy [43, 44], none described the difficult combination of ESRD, SLE and twin pregnancy.

SLE itself is a risk factor for every pregnancy. It is linked to the rare maternal death during pregnancy in dialysis patients [45], and LN activity correlates negatively with pregnancy outcomes. SLE pregnancies bear a high risk for hypertensive disorders, disease flares, placental insufficiency, but also fetal growth retardation and effects on the fetal cardiovascular system. Moreover, an augmented dialysis dose required to create optimal conditions for fetal development may reduce the uremic suppression of maternal (auto)immune activity, especially in SLE [10]. We followed management recommendations administering hydroxychloroquine to control disease activity and low-dose ASA due to its protective role against (pre)eclampsia. Apart from the low-dose corticosteroids given, e.g. azathioprine and calcineurin inhibitors are acceptable drugs to prevent flares during pregnancy. Moreover, in women with definite obstetric antiphospholipid syndrome - which was not the case in our patient - combination treatment with low dose ASA and heparin is recommended to decrease the risk of adverse pregnancy outcomes [46]. Escalation treatments such as IV immunoglobulins, increased doses of steroids, cyclophosphamide, or plasmapheresis were not required in our case.

Furthermore, multiple pregnancies themselves bear a 3 to 5-fold elevated risk of most maternal-fetal complications including (pre)eclampsia, intrauterine death, prematurity and more [47]. While some risks of twin pregnancies depend on their chorionic status, all twin gestations imply an increased risk for abnormal placentation. Notably, also twin pregnancies of healthy women typically conclude round 36 weeks of gestation [48].

In fact, a direct comparison of the outcome of our case with others is difficult due to the rare combination of the risk factors presented here. Certainly, if pregnancy in dialysis patients is combined with additional risks, compliance and strict adherence to the dialysis program is essential. The absence of such led to a less favorable progress even in a singleton pregnancy under similar conditions with severely premature ending at 28 WOG [44].

Moreover, the model of semi-inpatient dialysis with direct access to expertise in all relevant medical specialties was certainly optimal for preventing and managing complications associated with this high-risk pregnancy.

Our report supports the scientific consensus that a successful pregnancy even in dialysis patients at highest risk is feasible. Patients should not generally be discouraged to become pregnant or to delay pregnancy to a period after a desired kidney transplantation, but be informed about risks and required adaptations in renal replacement treatment and obstetric care. In our case, the multidisciplinary care finally achieved a good long-term result for mother and children.

## Data Availability

All data generated or analyzed are included in this published article.

## Abbreviations

ANA: Antinuclear antibodies
ASA: Acetylsalicylic acid
CKD: Chronic kidney disease
EPO: Erythropoietin
ESA: Erythropoiesis-stimulating agents
ESRD: End-stage renal disease
HD: Hemodialysis
ISN/RPS: International Society of Nephrology/Renal Pathology Society
IU: International units
MMF: Mycophenolate mofetil
NICU: Neonatal intensive care unit
LN: Lupus (glomerulo)nephritis
PD: Peritoneal dialysis
PRES: Posterior reversible encephalopathy syndrome
SD: Standard deviation
SLE: Systemic lupus erythematosus
WOG: Week(s) of gestation

## References

[1] Palmer BF. Sexual dysfunction in uremia. J Am Soc Nephrol. 1999;10(6):1381–8. 10361878 10.1681/ASN.V1061381

[2] Leão R, Sousa L, Azinhais P, Conceição P, Pereira BJ, Borges R, et al. Sexual dysfunction in uraemic patients undergoing haemodialysis: predisposing and related conditions. Andrologia. 2010;42(3):166–75. 10.1111/j.1439-0272.2009.00974.x 20500745

[3] Shahir AK, Briggs N, Katsoulis J, Levidiotis V. An observational outcomes study from 1966–2008, examining pregnancy and neonatal outcomes from dialysed women using data from the ANZDATA Registry. Nephrology (Carlton, Vic.). 2013;18(4):276–84. 10.1111/nep.12044 23441694

[4] Tangren J, Nadel M, Hladunewich MA. Pregnancy, end-stage renal disease. Blood Purif. 2018;45(1–3):194–200. 10.1159/000485157 29478065

[5] Piccoli GB, Cabiddu G, Daidone G, Guzzo G, Maxia S, Ciniglio I, et al. The children of dialysis: live-born babies from on-dialysis mothers in Italy–an epidemiological perspective comparing dialysis, kidney transplantation and the overall population. Nephrol Dial Transpl. 2014;29(8):1578–86. 10.1093/ndt/gfu092 24759612

[6] Luders C, Titan SM, Kahhale S, Francisco RP, Zugaib M. Risk factors for adverse fetal outcome in Hemodialysis pregnant women. Kidney Int Rep. 2018;3(5):1077–88. 30197974 10.1016/j.ekir.2018.04.013 PMC6127404

[7] Hoffman M, Sibai B. Dialysis in pregnancy: role of the underlying cause of renal failure on peripartum outcomes. Am J Perinatol. 2020;37(6):570–6. 31910463 10.1055/s-0039-3400307

[8] Parikh SV, Almaani S, Brodsky S, Rovin BH. Update on lupus nephritis: core curriculum 2020. Am J Kidney Dis. 2020;76(2):265–81. 32220510 10.1053/j.ajkd.2019.10.017

[9] Moroni G, Ponticelli C. Pregnancy in women with systemic lupus erythematosus (SLE). Eur J Intern Med. 2016;32:7–12. 27142327 10.1016/j.ejim.2016.04.005

[10] Althaf MM, Abdelsalam MS, Alfurayh OI. Lupus flares in two established end-stage renal disease patients with on-line hemodiafiltration during pregnancy - case series. Lupus. 2014;23(9):945–8. 24704775 10.1177/0961203314530487

[11] Sathiya R, Rajendran J, Sumathi S. COVID-19 and preeclampsia: overlapping features in pregnancy. Rambam Maimonides Med J. 2022;13(1). 10.5041/RMMJ.10464 PMC8798587 35089126

[12] Piccoli GB, Minelli F, Versino E, Cabiddu G, Attini R, Vigotti FN, et al. Pregnancy in Dialysis patients in the new millennium: a systematic review and meta-regression analysis correlating Dialysis schedules and pregnancy outcomes. Nephrol Dial Transpl. 2016;31(11):1915–34. 10.1093/ndt/gfv395 26614270

[13] Confortini P, Galanti G, Ancona G, Giongo A, Bruschi E, Lorenzini E. Full term pregnancy and successful delivery in a patient on chronic hemodialysis. Proc Eur Dial Transplant Assoc. 1971;(8):74–80.

[14] Okundaye I, Abrinko P, Hou S. Registry of pregnancy in Dialysis patients. Am J Kidney Dis. 1998;31(5):766–73. 9590185 10.1016/s0272-6386(98)70044-7

[15] Oliverio AL, Bragg-Gresham JL, Admon LK, Wright Nunes JA, Saran R, Heung M. Obstetric deliveries in US women with ESKD: 2002–2015. Am J Kidney Dis. 2020;75(5):762–71. 31785826 10.1053/j.ajkd.2019.08.029 PMC7183877

[16] Yang LY, Thia EWH, Tan LK. Obstetric outcomes in women with end-stage renal disease on chronic dialysis: a review. Obstet Med. 2010;3(2):48–53. 27582842 10.1258/om.2010.100001 PMC4989694

[17] Giatras I, Levy DP, Malone FD, Carlson JA, Jungers P. Pregnancy during dialysis: case report and management guidelines. Nephrol Dial Transpl. 1998;13(12):3266–72. 10.1093/ndt/13.12.3266 9870513

[18] Hou S. Conception and pregnancy in peritoneal Dialysis patients. Perit Dial Int. 2001;21(Suppl 3):S290–4. 11887838

[19] Wiles K, Chappell L, Clark K, Elman L, Hall M, Lightstone L, et al. Clinical practice guideline on pregnancy and renal disease. BMC Nephrol. 2019;20(1):401. 31672135 10.1186/s12882-019-1560-2 PMC6822421

[20] Bruno Vecchio RC, Del Negro V, Savastano G, Porpora MG, Piccioni MG. Dialysis Pregnancy: Overv Women. 2021;1(1):60–9.

[21] Hladunewich MA, Hou S, Odutayo A, Cornelis T, Pierratos A, Goldstein M, et al. Intensive Hemodialysis associates with improved pregnancy outcomes: a Canadian and united States cohort comparison. J Am Soc Nephrol. 2014;25(5):1103–9. 24525032 10.1681/ASN.2013080825 PMC4005313

[22] Cabiddu G, Castellino S, Gernone G, Santoro D, Giacchino F, Credendino O, et al. Best practices on pregnancy on dialysis: the Italian study group on kidney and pregnancy. J Nephrol. 2015;28(3):279–88. 25966799 10.1007/s40620-015-0191-3

[23] Arai H, Mori KP, Yokoi H, Mizuta K, Ogura J, Suginami K, et al. Intensified hemodialysis for complicated pregnancy in a primigravida with advanced maternal age: a case report with literature review focusing on appropriate hemodialysis management during pregnancy. Ren Replace Ther. 2020;6(1).

[24] Haase M, Morgera S, Bamberg C, Halle H, Martini S, Hocher B, et al. A systematic approach to managing pregnant Dialysis patients–the importance of an intensified haemodiafiltration protocol. Nephrol Dial Transpl. 2005;20(11):2537–42. 10.1093/ndt/gfi044 16115858

[25] Asamiya Y, Otsubo S, Matsuda Y, Kimata N, Kikuchi KAN, Miwa N, et al. The importance of low blood Urea nitrogen levels in pregnant patients undergoing Hemodialysis to optimize birth weight and gestational age. Kidney Int. 2009;75(11):1217–22. 19242506 10.1038/ki.2009.48

[26] Reddy SS, Holley JL. The importance of increased Dialysis and anemia management for infant survival in pregnant women on Hemodialysis. Kidney Int. 2009;75(11):1133–4. 19444267 10.1038/ki.2009.14

[27] Barua M, Hladunewich M, Keunen J, Pierratos A, McFarlane P, Sood M, et al. Successful pregnancies on nocturnal home Hemodialysis. Clin J Am Soc Nephrol. 2008;3(2):392–6. 18308997 10.2215/CJN.04110907 PMC2390936

[28] Kintiraki E, Papakatsika S, Kotronis G, Goulis DG, Kotsis V. Pregnancy-Induced hypertension. Horm (Athens). 2015;14(2):211–23. 10.14310/horm.2002.1582 26158653

[29] Hladunewich M, Schatell D. Intensive Dialysis and pregnancy. Hemodial Int. 2016;20(3):339–48. 27061443 10.1111/hdi.12420

[30] Tsakiridis I, Giouleka S, Arvanitaki A, Giannakoulas G, Papazisis G, Mamopoulos A, et al. Gestational hypertension and preeclampsia: an overview of National and international guidelines. Obstet Gynecol Surv. 2021;76(10):613–33. 34724074 10.1097/OGX.0000000000000942

[31] Collange O, Launoy A, Kopf-Pottecher A, Dietemann J-L, Pottecher T, Eclampsie. Ann Fr Anesth Reanim. 2010;29(4):e75–82. 20347562 10.1016/j.annfar.2010.02.021

[32] Chhabra S, Tyagi S, Bhavani M, Gosawi M. Late postpartum eclampsia. J Obstet Gynaecol. 2012;32(3):264–6. 10.3109/01443615.2011.639467 22369401

[33] Hinduja A. Posterior reversible encephalopathy syndrome: clinical features and outcome. Front Neurol. 2020;11:71. 32117030 10.3389/fneur.2020.00071 PMC7034490

[34] Hugonnet E, Da Ines D, Boby H, Claise B, Petitcolin V, Lannareix V, et al. Posterior reversible encephalopathy syndrome (PRES): features on CT and MR imaging. Diagn Interv Imaging. 2013;94(1):45–52. 22835573 10.1016/j.diii.2012.02.005

[35] Oliverio AL, Hladunewich MA. End-Stage kidney disease and Dialysis in pregnancy. Adv Chronic Kidney Dis. 2020;27(6):477–85. 33328064 10.1053/j.ackd.2020.06.001 PMC7781109

[36] Levy A, Fraser D, Katz M, Mazor M, Sheiner E. Maternal anemia during pregnancy is an independent risk factor for low birthweight and preterm delivery. Eur J Obstet Gynecol Reprod Biol. 2005;122(2):182–6. 16219519 10.1016/j.ejogrb.2005.02.015

[37] Sanchez-Gonzalez LR, Castro-Melendez SE, Angeles-Torres AC, Castro-Cortina N, Escobar-Valencia A, Quiroga-Garza A. Efficacy and safety of adjuvant Recombinant human erythropoietin and ferrous sulfate as treatment for iron deficiency anemia during the third trimester of pregnancy. Eur J Obstet Gynecol Reprod Biol. 2016;205:32–6. 27566219 10.1016/j.ejogrb.2016.08.004

[38] Sprenger-Mähr H, Zitt E, Kronbichler A, Cejna M, Lhotta K. A Hemodialysis patient with bone disease after pregnancy: a case report. BMC Nephrol. 2019;20(1):425. 31752733 10.1186/s12882-019-1603-8 PMC6873679

[39] Furaz-Czerpak KR, Fernández-Juárez G, La Moreno-de Higuera MÁ, Corchete-Prats E, Puente-García A. Martín-Hernández R. Pregnancy in women on chronic dialysis: a review. Nefrologia. 2012;32(3):287–94. 22508145 10.3265/Nefrologia.pre2012.Jan.11319

[40] Medeiros R, Pais MSJ, Freitas L, Moura P. Gravidez e Hemodiálise: A Propósito de Uma Gravidez Gemelar bem Sucedida. Acta Med Port. 2021;34(1):56–8. 33618795 10.20344/amp.11377

[41] Alix PM, Brunner F, Jolivot A, Doret M, Juillard L. Twin pregnancy in a patient on chronic haemodialysis who already had three pregnancies. J Nephrol. 2019;32(3):487–90. 30478508 10.1007/s40620-018-0555-6

[42] Da Alves Cunha IF, Marques Carreira CR. Successful twin pregnancy in Hemodialysis patient: multidisciplinary approach. Anesth Clin Res. 2017;08(08):1–4.

[43] Sivasuthan G, Dahwa R, John GT, Ranganathan D. Dialysis and pregnancy in end stage kidney disease associated with lupus nephritis. Case Rep Med. 2013;2013:923581. 10.1155/2013/923581 PMC3854106 24348579

[44] Ribeiro CI, Silva N. Pregnancy and Dialysis. J Bras Nefrol. 2020;42(3):349–56. 32776086 10.1590/2175-8239-JBN-2020-0028 PMC7657054

[45] Cabiddu G, Castellino S, Gernone G, Santoro D, Moroni G, Giannattasio M, et al. A best practice position statement on pregnancy in chronic kidney disease: the Italian study group on kidney and pregnancy. J Nephrol. 2016;29(3):277–303. 26988973 10.1007/s40620-016-0285-6 PMC5487839

[46] Andreoli L, Bertsias GK, Agmon-Levin N, Brown S, Cervera R, Costedoat-Chalumeau N, et al. EULAR recommendations for women’s health and the management of family planning, assisted reproduction, pregnancy and menopause in patients with systemic lupus erythematosus and/or antiphospholipid syndrome. Ann Rheum Dis. 2017;76(3):476–85. 27457513 10.1136/annrheumdis-2016-209770 PMC5446003

[47] Piccoli GB, Arduino S, Attini R, Parisi S, Fassio F, Biolcati M, et al. Multiple pregnancies in CKD patients: an explosive mix. CJASN. 2013;8(1):41–50. 23124785 10.2215/CJN.02550312 PMC3531652

[48] Gill P, Lende MN, van Hook JW. Twin births. In: StatPearls. Treasure Island (FL); 2021.

